# Students’ Perceptions of University Corruption in a Spanish Public University: A Path Analysis

**DOI:** 10.3389/fpsyg.2022.842345

**Published:** 2022-04-19

**Authors:** Martín Julián, Tomas Bonavia

**Affiliations:** ^1^Department of Psychology, Universidad Europea de Valencia, Valencia, Spain; ^2^Department of Social Psychology, Universitat de València, Valencia, Spain

**Keywords:** academic integrity, higher education, corruption, favoritism, bribery, fraud, embezzlement, path analysis

## Abstract

Most research on corruption in educational settings has focused on a cross-national and macro-level analysis; however, to our knowledge, few papers have sought to explore individual perceptions that explain corruption in higher education. The present research aimed to disentangle students’ predictors of corrupt intention in a Spanish public university. A total of 933 undergraduate, postgraduate, and Ph.D. students filled out an online survey measuring four corruption scenarios: favoritism, bribery, fraud, and embezzlement. Path analysis (PA) revealed that justifiability, risk perception, and perceived prevalence of corruption were significant factors in predicting corrupt intention. Moreover, willingness to report a corrupt act was predicted by corrupt intention, justifiability, and risk perception. Corrupt behavior is a complex phenomenon explained not only by peers’ behavior, but also by their individual justifications and perception of risk. Education is not free of corruption, and universities must address this urgent problem in order to avoid future economic, societal, and ethical problems.

## Introduction

Academics, politicians, and students worldwide have expressed their concern about the prevalence of corruption in education (Sabic-El-Rayess, 2016; Denisova-Schmidt, 2020; Sabic-El-Rayess and Heyneman, 2021), due to its harmful consequences for most university members (Chapman and Lindner, 2016; Maloshonok and Shmeleva, 2019; Denisova-Schmidt, 2020). In fact, previous research has highlighted the pervasiveness of corruption in the educational sector, from primary school to university, throughout the world (Heyneman, 2013; Denisova-Schmidt, 2017). Corrupt practices in universities are especially damaging because they produce a corrupt educational system that cannot afford to provide good services for students (Sabic-El-Rayess and Heyneman, 2021) and they also contribute to the rise of malpractices in their workplace when entering in the future job market (Ochulor, 2011).

Most of the research has analyzed corrupt behaviors from a cross-national perspective (Chapman and Lindner, 2016). To our knowledge, only a few papers have shown the relationship among students’ individual predictors when engaging in a corrupt behavior (Julian and Bonavia, 2020a,b). For example, a survey with 1,588 students showed that perceiving corruption as rampant among peers was associated with a rise in the willingness to offer a bribe (Shaw et al., 2015). Other authors (Čábelková and Hanousek, 2004) reached the same conclusion. Similarly, great tolerance toward fraud has been found in the educational sector (Gama et al., 2013). In a study conducted with 1,527 Portuguese university students, Gama et al. (2013) asked participants about their perceptions of corruption among their classmates, specifically for subtypes of fraud. Results pointed out that these kinds of behaviors were perceived as highly prevalent in this academic setting. In the same vein, in a study with 1,541 undergraduates, the willingness to get involved in a bribe was associated with perceiving corrupt behaviors as widely present (Liu and Peng, 2015).

However, it is crucial to understand that scientific literature on university corruption differs from academic integrity literature (Macfarlane et al., 2014). Academic integrity involves “multiple forms of academic deviance, including but not limited to test cheating, plagiarism, and inappropriate collaboration” (Kisamore et al., 2007, p. 382), whereas corruption is commonly understood in the academic literature as “the abuse of entrusted power for private gain” (Transparency International, 2009, p. 14). With regard to the actors involved in corrupt activities at the university, they can be students, administrators, private suppliers, and the teaching staff (Hallak and Poisson, 2007; Denisova-Schmidt, 2017, 2020). University corruption ranges from paying a bribe to getting admitted into an academic program to assigning public positions through favoritism (Hallak and Poisson, 2007; Denisova-Schmidt, 2017, 2020). Furthermore, it has been observed that corruption appears in different forms in the educational system: in its educational functions, in supplying goods and services, in the lack of correct conduct among professionals, and in its management of property and taxes. Accordingly, previous research (Sabic-El-Rayess, 2016) has stated that “knowing more about typologies of educational corruption and quantifying the corruption or the perception of it is a salient and still evolving research area in education” (p. 20). As early research (Hallak and Poisson, 2007, p. 57) states, favoritism is “a mechanism of power abuse implying privatization and a highly biased distribution of state resources”; bribery is “a payment (in money or in kind) given or taken in a corrupt relationship”; fraud is “an economic crime that involves some kind of trickery, swindling, or deceit”; and embezzlement is “the theft of public resources by public officials.” Given that fraud and bribery can be both financial and non-financial in nature (Hallak and Poisson, 2007), they will be perceived differently depending on their manifestation. The present study addresses a non-financial form of these corrupt behaviors because they are more likely to occur at the university level and they are highly extended, as it can be observed when obtaining admission to a degree program or manipulating students’ marks to obtain some non-financial benefits such as gaining workforce to get tasks done or producing research at a minimum cost (Hallak and Poisson, 2007; Denisova-Schmidt, 2020).

Based on these considerations, the present paper aims to study the students’ predictors of corrupt intention in a Spanish public university by developing a theoretical model. The current situation of the Spanish university system is largely characterized by strong competitiveness among universities (Rincón and Barrutia, 2017). There are 84 universities in Spain: 50 are public, and 34 are private (MECD, 2016). It was chosen a Spanish public university that has around 55,000 students and 3,300 academic staff members. This university offers degrees in a wide range of academic fields: arts and humanities, engineering, health sciences, sciences, and social sciences. Students have access to different degrees, official masters, and Ph.D. programs. Although academic dishonesty has been analyzed in Spain (Comas-Forgas and Sureda-Negre, 2016), studies on individual predictors of Spanish university corruption are still scarce (Julian and Bonavia, 2020a,^2021^). Furthermore, nearly two-thirds of Spanish citizens rated corruption as a major problem in their country (Transparency International, 2016).

In our study, four types of corruption behaviors will be evaluated using the same theoretical model: favoritism, bribery, fraud, and embezzlement. To our knowledge, this is the first paper to study individual predictors of university corruption in a Spanish context while proposing a theoretical model to understand its diverse manifestations (Johnsøn and Mason, 2013; Denisova-Schmidt, 2016; Julian and Bonavia, 2020b). Similar studies have been published (see, e.g., Julian and Bonavia, 2021), but they did not include a reporting intention variable (whistleblowing), and they used a different methodological analysis of the results.

### Hypotheses

Given that corrupt activities are risky, early research has shown that corrupt intention is partially predicted by risk perceptions, so that there is a negative association between these two variables (Berninghaus et al., 2013; Julian and Bonavia, 2021). In fact, most people are unable to estimate the probability of risk situations accurately, and they think they will not be discovered (Kahneman and Tversky, 1973; Frederick, 2005). In addition, some authors have shown that not having information about what other people are doing (corrupt practices) operates as a powerful deterrent to participation in such acts (Ryvkin and Serra, 2012; Berninghaus et al., 2013). Power asymmetry plays a fundamental role in magnifying the effect of uncertainty, such that the greater the power asymmetry, the greater the influence of the perception of uncertainty on the people involved (Ryvkin and Serra, 2012). This scenario is frequent when students and professors are interacting with each other. Therefore, the first hypothesis will be:

*H*_1_ Risk perception negatively predicts corrupt intention.

The pervasiveness of corrupt behaviors produces an embedding of these conducts (Shaw et al., 2015; Maloshonok and Shmeleva, 2019). Scientific research has pointed out that perceiving high levels of corruption contributes to generating a belief that it is difficult to be caught when performing an act of this magnitude (Dong et al., 2012). Moreover, it encourages distorted beliefs about dishonesty, destroys social capital (trust), reduces honest behaviors, contributes to manipulating what is understood as a social norm, and generates a breeding ground for the emergence of future illegal actions (Kallgren et al., 2000; Gino et al., 2009; Dong et al., 2012). Studies have shown (Cialdini et al., 1990; Reno et al., 1993) that “injunctive norms” (allowability of an act) and “descriptive norms” (the extent to which people participate in an activity) are crucial in disentangling corrupt behavior. Injunctive norms stir negative feelings when deciding to participate in a corrupt scenario, whereas descriptive norms help to justify corrupt behavior (Köbis et al., 2015). Therefore, the second hypothesis will be:

*H*_2_ The perceived prevalence of corruption positively predicts corrupt intention.

Closely related to this hypothesis, ethical judgment has been found to have an influence on people’s attitudes toward corruption (Jones, 1991; Julian and Bonavia, 2020a). Many situations are evaluated within a moral framework, and so justifications play a key role in influencing people’s decision to take part (or not) in a corrupt activity (Gino et al., 2009). People are able to rationalize dishonest behaviors if these behaviors benefit them, and people can be honest or dishonest based on the characteristics of the situation (Mazar et al., 2008; Gächter and Schulz, 2016; Shalvi, 2016). For example, ethical assessment modifies the probability of carrying out an unethical act and, putting aside the quality of a lie, people tend to use self-justifications to lie more (Shalvi et al., 2011). In fact, justifying an unethical behavior reduces the perception of the act as being unethical (Shalvi et al., 2011). The “self-concept maintenance theory” posits that people reduce their cognitive dissonance by relying on strategies that modify their values so that they are congruent with their dishonest behavior (Mazar et al., 2008). One of the most powerful strategies to protect the self-concept is self-justification (Shalvi et al., 2015). Before engaging in corruption, people tend to elaborate moral justifications to excuse ethical dissonance and maintain a positive self-concept. After engaging in corruption, they tend to elaborate moral justifications to compensate for harmful consequences (Shalvi et al., 2015). Put differently, having to decide whether a potential benefit (in the present study, engaging in a corrupt activity) offsets the ethical cost of acting dishonestly requires people to elaborate justifications to lessen such costs (Wenzel et al., 2017).

Therefore, the third hypothesis will be:

*H*_3_ Justifiability of corruption positively predicts corrupt intention.

When trying to decide whether to blow the whistle, people take ethical issues into account (Dungan et al., 2015). Scientific literature has shown that moral values play a crucial role in reporting corrupt behavior, and situational factors (e.g., the organization’s encouragement to report dishonest activities) complement individual factors in explaining the justifiability of corrupt intention (Moore, 2008, 2015; Julian and Bonavia, 2020a). In other words, people will conceptualize wrongdoing as justifiable if they think it is not harmful to the organization. Moreover, the perception of values such as nepotism, loyalty and economic gains and moral values influence people’s attitude toward corrupt activities (Park and Lewis, 2019; Tu et al., 2020). When normalizing favoritism or excessively valuing material gains, people tend to tolerate corrupt acts and refrain from taking part in anti-corruption practices such as reporting or whistleblowing (Tu et al., 2020). Therefore, the fourth hypothesis will be:

*H*_4_ Justifiability of corruption negatively predicts reporting intention.

Along with personal characteristics such as extraversion or feeling an internal locus of control (Bjørkelo et al., 2010), corrupt intention has been found to play a role in explaining reporting intention. As some authors have argued (Cialdini et al., 1995), people tend to pursue consistent attitudes in everyday life. Classical research on psychology has shown that reducing cognitive dissonance is a crucial task for people (Festinger, 1957). Changing one’s attitude about corrupt intention implies deciding whether or not to blow the whistle, due to the conflict originated by this dilemma. In the same vein, there are psychological mechanisms that allow people to reformulate their own attitudes or behaviors to maintain internal moral standards (Mazar et al., 2008). Therefore, the fifth hypothesis will be:

*H*_5_ Corrupt intention negatively predicts reporting intention.

As mentioned above, whistle-blowers are prone to reporting corrupt behavior in organizations that support whistleblowing (Mesmer-Magnus and Viswesvaran, 2005) because the threat of retaliation influences people’s attitudes toward whistleblowing. Particularly, if people perceive a high risk of being caught when observing peers engaging in corrupt activities, they will also feel that reporting corrupt activities is safe and that they will not be punished for doing so (Mesmer-Magnus and Viswesvaran, 2005; Oelrich, 2021). A retaliatory climate emerges when risk perception is low and there is a widely assumed norm that corrupt acts are allowed, so many whistleblowers are often punished once they have reported misconduct (Oelrich, 2021). What is more, people are prone to refrain from blowing the whistle (again) if they interpret the situation as characterized by negative consequences and if they perceive that the moral costs of doing so are low (Oelrich, 2019; Park and Lewis, 2019). Therefore, the sixth hypothesis will be:

*H*_6_ Risk perception positively predicts reporting intention.

Research has shown that, in circumstances where moral issues are salient, risk perceptions are conceptualized differently (Eckensberger et al., 2001). This idea challenges accepted conceptions of rational decision-making theories, which exclude moral or ethical standards. Research on risk analysis has shown that risk is evaluated from an analytic approach, which draws on a logical and rational system of deliberation, and from an affective approach, which consists of an intuitive and fast deliberation system (Slovic et al., 2004). The latter system highlights the quality of ‘goodness’ or ‘badness’ and stresses the characterization of a stimulus as negative or positive. Subsequently, risk perception and justifiability will be strongly related when facing a risky situation. Therefore, the seventh hypothesis will be:

*H*_7_ Risk perception is negatively correlated with justifiability of corruption.

## Materials and Methods

### Participants

The sample consisted of 933 Spanish university students (Mage = 24.92 years, SD = 8.78) who answered the questionnaires voluntarily: 75.3% were undergraduate students, 16.6% were postgraduate students, and 8.1% were Ph.D. students. Regarding sex, 67.1% were women, and 32.9% were men (overall, 60% of the students in this university are women). Descriptive analysis showed that only 5.5% of these students were members of a research group.

### Instruments

Corruption is hard to measure because of its secrecy and illegality. Although there is divergence in the corruption literature about their advantages and disadvantages, hard data (objective approach) are difficult to obtain and tend to be more accurate, whereas perception indices are easier to implement and tend to be less accurate (Johnsøn and Mason, 2013; Charron, 2016). Given that the perception of corruption and previous experience with such acts are associated (Charron, 2016), information was gathered by considering the perceived prevalence of corruption among the participants. Some international organizations -e.g., Transparency International- use these instruments to collect their data (Martinez, 2016).

Based on previous research (León et al., 2013; Liu et al., 2017; Chen et al., 2021), a ‘vignette methodology’ was applied, which consists of a hypothetical vignette description -in this case, corrupt activities-. These studies implemented an online survey using these anchoring vignettes, as in the present study. Asking participants about specific corrupt practices is useful to obtain data about the subjective scale individuals are using when answering questions about corruption. As early research has shown, “the methodology involves a correction of the individual assessment based on a general scale of corruption for the responses to the hypothetical scenarios defined in the vignettes that lead to a more accurate assessment of corruption” (León et al., 2013, p. 979). Four vignettes were employed to analyze favoritism, bribery, fraud, and embezzlement (see ‘Appendix’ for their description). An expert panel from the university where the present study was conducted helped develop scenarios of corrupt activities. Expert assessment involved ratings of the clarity, readability, and realism. Scenarios were revised according to the panel’s suggestions.

Once participants had read each vignette, they answered five questions. Originally, both the vignettes and the items were presented to students in Spanish.

#### Perceived Prevalence of Corruption

Given scientific literature on perception of corruption among peers (Dong et al., 2012; Transparency International, 2016; Liu et al., 2017; Julian and Bonavia, 2021), a single-item measure was used for perceived prevalence of corruption: “Please, mark how often you think this kind of situation happens in this university?” Response scale ranges from 1 (Never) to 5 (Always).

#### Justifiability

Previous studies stated that assessing justifiability using a single item result in similar outcomes when compared to laboratory experiments (Cummings et al., 2009). Based on previous corruption research (Dong et al., 2012; Liu et al., 2017; Chen et al., 2021; Julian and Bonavia, 2021), a single-item measure was used for justifiability of corruption: “To what extent would you consider it justifiable to accept a proposal like this one?” Response scale ranges from 1 (Unjustifiable) to 5 (Totally justifiable).

#### Risk Perception

Considering that risk perception can be accurately measured by a single question (Ganzach et al., 2008), and that corrupt intention is also effectively predicted by this variable when compared to other variables such as risk attitude, (Berninghaus et al., 2013), a single-item measure was used for risk perception: “To what extent do you think that, if you accepted, your class-mates would find out?” Response scale ranges from 1 (There is no risk) to 5 (Extreme risk).

#### Corrupt Intention

Different scholars have demonstrated that corrupt intention is a valid instrument to study corruption (Dong et al., 2012; León et al., 2013; Julian and Bonavia, 2020a; Chen et al., 2021). The question to measure corrupt intention was the following one: “If this situation happened to you in real life, how likely is it that you would accept this proposal?” Response scale ranges from 1 (I would not accept it at all) to 5 (I would certainly accept it).

#### Reporting Intention

Based on an anonymous channel to capture whistle-blowing intention (Kaplan et al., 2009), a single-item measure was used for reporting intention: “If this situation happened to your classmate and you could do something anonymously, what would you do?” Response scale ranges from 1 (I would not do anything) to 5 (I would report this situation).

### Procedure

After approval by the Vice-Chancellor of Research of the University, an online survey (written in Spanish) was delivered to all the students by utilizing the university database. This means that all the students received the online survey at their university email address. They did not have to register in an account or use any login details. Participants’ anonymity and confidentiality were guaranteed during data collection. After two weeks of data collection (during November), the questionnaire was closed in order to transfer the data into the corresponding software.

### Data Analysis

**FIGURE 1:**
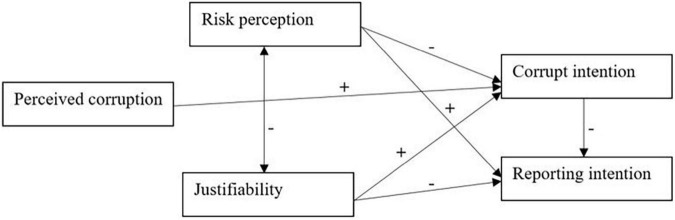
Hypothesized model.

Structural Equation Modeling (SEM) was used –specifically, a path analysis (maximum likelihood estimation)- to test the proposed model in Figure 1. Analyses were carried out using Mplus version 7 (Muthén and Muthén, 2012). Assumptions of normality were checked. Considering the criteria proposed by some authors (Hu and Bentler, 1999), the fit of the model was assessed with the following indexes: Chi-Square (χ2), Comparative Fit Index (CFI), Root Mean Square Error of Approximation (RMSEA), and Standardized Root Mean Residual (SRMR).

## Results

As Table 1 shows, students perceived favoritism as more extended than bribery [*t*(932) = 17.63, *p* < 0.001, *d* = 0.57], fraud [*t*(932) = 7.65, *p* < 0.001, *d* = 0.25] and embezzlement [*t*(932) = 12.10, *p* < 0.001, *d* = 0.39]. It is worth reminding that the participants were asked about perceived prevalence of corruption, not about their own experiences. These results are complemented by the responses on justifiability; that is, students also thought it was more justifiable to engage in favoritism than engaging in embezzlement [*t*(932) = 25.69, *p* < 0.001, *d* = 0.84]. However, there was not significant differences between favoritism’s justifiability and bribery’s justifiability [*t*(932) = 0.85, *p* = 0.391, *d* = 0.02], or between favoritism’s justifiability and fraud’s justifiability [*t*(932) = 0.74, *p* = 0.454, *d* = 0.02].

**TABLE 1:** Mean and standard deviation for model’s variables.

Variable	Model						
	Favoritism		Bribery		Fraud		Embezzlement	
	M	SD	M	SD	M	SD	M	SD
Perceived corruption	3.07	1.16	2.38	1.05	2.80	1.08	2.61	2.26
Risk perception	3.56	1.10	2.71	1.17	2.92	1.12	3.64	1.16
Justifiability	3.17	1.20	3.12	1.37	3.13	1.21	1.93	1.16
Corrupt intention	3.52	1.20	3.58	1.40	3.31	1.28	2.20	1.32
Reporting intention	2.83	1.32	2.20	1.34	2.51	1.32	3.22	1.48

*Response scale ranges from 1 to 5 in all measures.*

Regarding the riskiest corrupt behavior, students perceived embezzlement as riskier than bribery [*t*(932) = 19.30, *p* < 0.001, *d* = 0.63] and fraud [*t*(932) = 16.27, *p* < 0.001, *d* = 0.53], but there was not differences between embezzlement and favoritism based on the risk perception [*t*(932) = 1.53, *p* = 0.125, *d* = 0.05].

Participants showed a higher reporting intention when they read the vignette about embezzlement rather than when they read the vignettes of favoritism [*t*(932) = 7.19, *p* < 0.001, *d* = 0.23], bribery [*t*(932) = 18.77, *p* < 0.001, *d* = 0.61] and fraud [*t*(932) = 13.96, *p* < 0.001, *d* = 0.45].

Finally, students were more prone to commit a behavior of favoritism than a behavior of fraud [*t*(932) = 5.28, *p* < 0.001, *d* = 0.17] and embezzlement [*t*(932) = 27.71, *p* < 0.001, *d* = 0.90]. However, no significant differences were found between favoritism and bribery regarding behavioral intention [*t*(932) = −1.35, *p* = 0.177, *d* = −0.04].

Except chi-square (χ2) and RMSEA, Table 2 shows an acceptable fit for the proposed model in all the corrupt behaviors. Early research (Kline, 2011) proposes that observed values of χ2 increase along with sample size –as it happened in the current study–. A large sample size can produce a failure in the chi-square test (e.g., finding a significant χ2 value), even when differences between observed and predicted co-variances are minimal. In the case of RMSEA, previous research (Mulaik, 2009) has shown that it is affected by the sample size, becoming larger as the sample size increases. In addition, the RMSEA index penalizes complexity in smaller models with few variables (Breivik and Olsson, 2001), such as the current hypothesized models.

**TABLE 2:** Fit statistics of corruption models.

Model	χ ^2^	*df*	CFI	RMSEA	SRMR
Favoritism	19.01***	3	0.98	0.07	0.03
Bribery	39.98***	3	0.96	0.11	0.04
Fraud	39.96***	3	0.96	0.11	0.04
Embezzlement	78.06***	3	0.93	0.16	0.06

Some authors (Hu and Bentler, 1999) suggest that an acceptable fit of a model should be based on CFI ≥ 0.95; RMSEA ≤ 0.06 and SRMR ≤ 0.08. ***p < 0.001.

Path analyses were conducted to verify the proposed hypotheses. As Table 3 indicates, all the paths showed significant differences, except the path from perceived prevalence of corruption to corrupt intention in bribery and fraud, and the path from risk perception to reporting intention in favoritism. Corrupt intention decreased as students’ risk perception increased in all the corrupt behaviors, supporting the first hypothesis. Students’ corrupt intention was positively associated with their perception of corruption in the favoritism and embezzlement behaviors, but this association was not found in the bribery and fraud behaviors. Based on these results, the second hypothesis was confirmed in two of the corruption scenarios. Corrupt intention was positively associated with justifiability, and justifiability negatively predicted reporting intention in all the corrupt behaviors, supporting the third and fourth hypotheses, respectively. As proposed in the fifth hypothesis, as corrupt intention increased, students’ reporting intention decreased. Regarding the sixth hypothesis, as risk perception increased, participants’ intention to report also increased in all the corrupt behaviors, except favoritism. Finally, risk perception correlated negatively with justifiability in all the corrupt behaviors, as proposed in the seventh hypothesis. In all the corrupt behaviors, justifiability was the best predictor of corrupt intention, whereas the perceived prevalence of corruption hardly explained corrupt intention.

**TABLE 3:** Standardized coefficients of path analysis for corruption models.

Path		Model			
Predictor	Criterion	Favoritism	Bribery	Fraud	Embezzlement
Risk perception	Corrupt int.	−0.17***	−0.07***	−0.09***	−0.07***
Perceived corruption	Corrupt int.	0.04*	0.02	0.02	0.06**
Justifiability	Corrupt int.	0.64***	0.73***	0.72***	0.75***
Risk perception	Reporting int.	0.03	0.11***	0.15***	0.07*
Justifiability	Reporting int.	−0.17***	−0.22***	−0.25***	−0.19***
Corrupt intention	Reporting int.	−0.25***	−0.24***	−0.09*	−0.31***
**Correlation of predictors**				
Risk perception	Justifiability	−0.17***	−0.25***	−0.21***	−0.13***

*p < 0.05; **p < 0.01; ***p < 0.001.

## Discussion

The present research focused on studying students’ perceptions of corrupt intention at the university level. Specifically, a theoretical model was tested for four common corrupt behaviors: favoritism, bribery, fraud, and embezzlement (Hallak and Poisson, 2007). According to early research (Johnsøn and Mason, 2013), corrupt behavior should be analyzed by studying its diverse manifestations, and this suggestion has been followed in the present research. Additionally, university students were sampled to analyze university corruption in accordance with the current objectives. In order to achieve greater external validity and coherence between the sample and the variables, vignettes were constructed intentionally to recreate a realistic and credible environment for the students (Denisova-Schmidt, 2016).

Regarding embezzlement, the results confirmed all the proposed hypotheses. Analyses showed that risk perception correlated negatively with justifiability, and that corruption intention decreased as perception of risk increased, coinciding with previous literature suggesting that people base their judgment of an activity on what they think and feel about it (Gino et al., 2009; Berninghaus et al., 2013; Julian and Bonavia, 2021). If they feel something is wrong, they will conceptualize the risks as high and the benefits as low, and vice versa (Slovic et al., 2004). Furthermore, it was found that as risk perception increased, participants’ intention to report corruption also increased. Contextual factors such as perceived costs or risk perception influence personal determination to report corrupt behavior, and so a high-risk perception of being caught will result in a greater tendency to report corrupt intention, due to the perceived safety of the observer (Mesmer-Magnus and Viswesvaran, 2005; Oelrich, 2021). This may be due to the perception that reporting activities is safe and that there is no punishment for doing so, that is, people carry out an analysis of cost-benefits before making a decision on blowing the whistle based on what happens to their peers when engaging in reporting activities (Mesmer-Magnus and Viswesvaran, 2005). In this vein, Werber and Balkin (2010) stated that people weigh all the possible scenarios before engaging in dishonest activities, that is, they compare the gains (rewards or incentives) to the losses (performance assessment or getting fired). Therefore, the co-workers will evaluate the consequences of such activity to determine if it is worthwhile whistleblowing.

In the case of favoritism and embezzlement, the results also showed that, as students’ perception of corruption increased, their corrupt intention also increased, as in previous literature (Dong et al., 2012; Maloshonok and Shmeleva, 2019; Julian and Bonavia, 2021). If people acknowledge that a situation is highly corrupt, they will have a greater tendency to engage in corrupt behaviors, due to a phenomenon called the “contagion effect” (Gino et al., 2009). Observing dishonest acts all around them makes people miscalculate their own chances of getting caught, and they are more likely to modify their perceptions of social norms (Cialdini et al., 1990).

The current results also provide evidence for the role of justifiability. Justifiability positively predicted corrupt intention and negatively predicted reporting intention. In the former case, the explanation might come from early research showing that, once justifications for dishonest conduct are collected, people are more motivated to take part in such actions (Gino et al., 2009). In other words, ethical rationalizations contribute to reducing moral harm when someone behaves dishonestly (Shalvi et al., 2015). In the same vein, as the aforementioned research showed, reporting corruption depends on people’s view of fairness and loyalty values, choosing fairness as the priority if they decide to blow the whistle (Dungan et al., 2015). Ethical issues are at stake when trying to decide whether or not to report a corrupt activity, and a perception of harm to third parties increases the probability of reporting wrongdoing (Moore, 2008).

Finally, the results showed that, as corrupt intention increases, participants’ intention to report corruption decreases. Given the influence of ethical issues on a key decision such as taking part in corruption, reporting intention (whistleblowing) will depend on ethical rationalizations made by a person in that situation (Shalvi et al., 2015). As stated in early research (Cialdini et al., 1995), people seek to create a positive self-concept by reducing cognitive dissonance (Mazar et al., 2008). Therefore, our findings are in line with research in this area (Dong et al., 2012).

Regarding bribery and fraud, our findings supported all the hypotheses, except the second one. Additionally, the present outcomes suggest a weak association between perceived prevalence of corruption and corrupt intention, which means that perceiving corrupt behaviors all around is not a powerful factor in someone’s intention to engage in corrupt practices. On the other hand, it is also possible that students were influenced by the moral saliency of the vignettes, as suggested by early research on moral psychology (Jones, 1991; Moore, 2008). People evaluate moral issues when making decisions, not only concluding that a decision implies moral values, but also paying attention to specific moral characteristics of the situation (Jones, 1991). Thus, it is likely that students tended to focus on moral issues when deciding whether to accept a corrupt proposal, rather than paying attention to the perceived prevalence of corruption.

The present research has some limitations. First, vignettes have higher external validity when it comes to drawing conclusions from a study, but they are also limited to a very specific situation (or situations). Due to its methodological nature (cross-sectional and field survey), the present study presents some threats to both its external and internal validity. In the former case, there could be uncontrolled variables that may be influencing the final outcomes (Roe and Just, 2009), such as living in a household that actively tolerates high levels of corruption. In the latter case, relying on a single-case study of a public institution may not be enough to bring general conclusions about the prevalence and explanations of corruption in academia. However, due to its relevance and its structural characteristics, the present results could be useful to understand corrupt dynamics in an educational institution. It is also worth noting that all the variables were measured with single items, which undermines the robustness and reliability of the present study. This limitation could be solved by developing or implementing standardized instruments to measure each variable in future studies. Second, it was studied the students’ point of view about university corruption, but the perceptions of administrators, private suppliers, or teaching staff was not considered, in line with early research (Hallak and Poisson, 2007; Denisova-Schmidt, 2016). Third, the present sample was mainly composed of women, and some studies on corruption have shown that women behave in a less corrupt way than men (Ayal et al., 2015). Social desirability or previous experience with corrupt activities could be influencing the present outcomes as well. There should be also considered the structure of Spanish universities, given that corrupt activities are embedded in a society –national and regional context– and influenced by cultural factors (Charron, 2016; Rincón and Barrutia, 2017). Additionally, it is necessary to carry out further experimental research to show possible casual relationships among key variables related to university corruption, as previous research has pointed out (Denisova-Schmidt, 2016). Although making use of a path analysis is useful to elaborate explanations of a social phenomenon, this kind of statistical analysis is not addressed to establish causes between variables (Jeon, 2015). Previous research (Jeon, 2015) has also stated that there are several limitations of assumptions in a path analysis:

(1) Relations among variables in the model are linear, additive, and causal. (2) Each residual is not correlated with variables that precede it in the model. (3) There is one way causal flow. That is reciprocal causation between variables is ruled out. (4) The variables are measured on an interval scale. (5) The variables are measured without error. All these assumptions are hard to be satisfied in social science (p. 1,638).

It is also important to highlight that the present study sought to understand corrupt practices in a public university, but there is still a gap in the scientific literature regarding possible differences between public and private universities. Future research could take this lack of data into account to reduce this gap.

The results indicate that an increase in moral/ethical cues among university members is necessary to combat corruption, due to the magnitude of justifiability in corrupt intention. Promoting more transparent and clearer administration procedures could help to foster a perception of high risk in people and, consequently, deter them from performing dishonest acts. In order to promote these policies, there must be anonymous whistle-blowing sources to favor corruption reports at the university. In a similar vein, previous studies (Heyneman, 2013) encourage universities to protect university staff from retaliation and provide them with secure and reliable ways to report activities involving wrongdoing. In fact, some authors (Ayal et al., 2015) have worked on a three-principle guide for designing effective policies to reduce dishonest behavior. Considering the present results, these guidelines could be helpful in tackling corruption in higher education: “reminding,” which means introducing subtle cues designed to increase moral salience and decrease dishonesty justifications; “visibility,” which is related to social monitoring mechanisms such as anonymity (or confidentiality of private information, if not possible) restrictions or fostering peer monitoring; and “self-engagement,” which aims to encourage people to act morally by increasing their positive self-image.

Previous research has shown that it is necessary to study different types of corruption in order to comprehend this phenomenon in depth (Johnsøn and Mason, 2013; Julian and Bonavia, 2020b). In fact, corruption (and its typologies) in the education sector has been poorly studied for decades (Hallak and Poisson, 2007; Julián and Bonavia, 2017; Denisova-Schmidt, 2020). The present paper aimed to challenge this issue by analyzing favoritism, bribery, fraud, and embezzlement in an educational setting. It is proposed that perception of risk and justifiability influence students’ decisions to engage in corrupt activities and reporting intentions at the university.

Most of all, the results reveal that the education sector is not free of corruption, and universities must face this urgent problem in order to avoid future economic, societal, and ethical problems (Sabic-El-Rayess and Heyneman, 2021). A corrupt university results in corrupt members and a pervasive conduct that reinforces unethical behavior and penalizes a curriculum based on merit and personal effort (Chapman and Lindner, 2016; Denisova-Schmidt, 2020). This is what the present research aimed to disentangle.

## Data Availability Statement

The original contributions presented in the study are included in the article/Supplementary Material, further inquiries can be directed to the corresponding author/s.

## Ethics Statement

The studies involving human participants were reviewed and approved by Comité Ético de Investigación en Humanos (University of Valencia). The patients/participants provided their written informed consent to participate in this study.

## Author Contributions

All authors listed have made a substantial, direct, and intellectual contribution to the work, and approved it for publication.

## Conflict of Interest

The authors declare that the research was conducted in the absence of any commercial or financial relationships that could be construed as a potential conflict of interest.

## Publisher’s Note

All claims expressed in this article are solely those of the authors and do not necessarily represent those of their affiliated organizations, or those of the publisher, the editors and the reviewers. Any product that may be evaluated in this article, or claim that may be made by its manufacturer, is not guaranteed or endorsed by the publisher.

## Appendix

Description of the hypothetical scenarios.

Next, you will see a hypothetical situation based on real experience. Imagine that you are the one who has to make the decision, and then answer the following questions. (This introduction was presented at the beginning of each scenario).

*Scenario 1 (favoritism)*

You recently finished your university degree, and you are collaborating with a professor on a research project. One day, your professor tells you that there will be a public job in the project you are working on. Your professor tells you that he/she has thought of you for that job because you work really well. He/she asks you not to talk about this issue with your teammates while going through the formal selection process, but you will be chosen in the end.

*Scenario 2 (bribery)*

You are asking for a university scholarship. However, your grade average has gone down because of a subject in which you did not do well on the exam. After the exam, you go for a tutorial session because you want to know how to improve your grade. You describe your poor economic situation to your professor and you ask him/her to raise your grade because you really need that scholarship. After thinking carefully, your professor tells you to do some research work for him/her in exchange for the grade. He/she warns you that you cannot say anything about it to your classmates because it is an exceptional situation that he/she cannot offer everyone.

*Scenario 3 (fraud)*

You have just finished your degree and you want to enroll in a postgraduate degree. You find a good one and very prestigious, but it is difficult to be accepted. Moreover, you find out that one of your degree professors teaches a subject in the postgraduate degree. You decide to tell him/her that you are really interested in being admitted to the postgraduate degree program. He/she mentions that he/she knows the selection committee members. He assures you that he/she is able to facilitate your admission. In return, he/she asks you to collaborate on a research project in which you are not interested.

*Scenario 4 (embezzlement)*

After a few months of collaborating on a research project with a professor, the opportunity arises to attend a conference in the USA for 2 days in the summer. Although the conference hardly focuses on your research interests, your professor proposes spending public teaching funds to pay the expenses for both of you during the two days of the conference, plus a stay lasting more 8 days.
